# Going Coastal: Shared Evolutionary History between Coastal British Columbia and Southeast Alaska Wolves (*Canis lupus*)

**DOI:** 10.1371/journal.pone.0019582

**Published:** 2011-05-04

**Authors:** Byron V. Weckworth, Natalie G. Dawson, Sandra L. Talbot, Melanie J. Flamme, Joseph A. Cook

**Affiliations:** 1 Faculty of Environmental Design, University of Calgary, Calgary, Alberta, Canada; 2 Department of Biology and Museum of Southwestern Biology, University of New Mexico, Albuquerque, New Mexico, United States of America; 3 U.S. Geological Survey, Alaska Science Center, Anchorage, Alaska, United States of America; 4 National Park Service, Yukon-Charley Rivers National Preserve, Fairbanks, Alaska, United States of America; Smithsonian Institution National Zoological Park, United States of America

## Abstract

**Background:**

Many coastal species occupying the temperate rainforests of the Pacific Northwest in North America comprise endemic populations genetically and ecologically distinct from interior continental conspecifics. Morphological variation previously identified among wolf populations resulted in recognition of multiple subspecies of wolves in the Pacific Northwest. Recently, separate genetic studies have identified diverged populations of wolves in coastal British Columbia and coastal Southeast Alaska, providing support for hypotheses of distinct coastal subspecies. These two regions are geographically and ecologically contiguous, however, there is no comprehensive analysis across all wolf populations in this coastal rainforest.

**Methodology/Principal Findings:**

By combining mitochondrial DNA datasets from throughout the Pacific Northwest, we examined the genetic relationship between coastal British Columbia and Southeast Alaska wolf populations and compared them with adjacent continental populations. Phylogenetic analysis indicates complete overlap in the genetic diversity of coastal British Columbia and Southeast Alaska wolves, but these populations are distinct from interior continental wolves. Analyses of molecular variation support the separation of all coastal wolves in a group divergent from continental populations, as predicted based on hypothesized subspecies designations. Two novel haplotypes also were uncovered in a newly assayed continental population of interior Alaska wolves.

**Conclusions/Significance:**

We found evidence that coastal wolves endemic to these temperate rainforests are diverged from neighbouring, interior continental wolves; a finding that necessitates new international strategies associated with the management of this species.

## Introduction

Evaluating phylogeographic patterns across multiple species can aid in deciphering the historical processes that drive community assemblage and species diversification throughout regions of interest [1]. For example, geographic isolation due to glacial vicariance can fragment species into genetically disjunct populations. Over time, these populations may diverge into distinct evolutionary lineages. In North America, the regional fauna along the North Pacific Coast exhibit phylogeographic patterns consistent with long-term isolation due to large scale historical climatic events [2], with a growing number of distinctive and often endemic “coastal” lineages identified across an array of organisms [3]–[7]. A clear view of the biogeographic history and spatial distributions of these lineages is important for assessing and maintaining genetic diversity in this distinctive biome; however, most species remain poorly documented, precluding the rigorous application of scientific knowledge to objective management.

Recent molecular studies of wolves supported both phylogeographic and ecological mechanisms for diversification of wolves (*Canis lupus*) in western North America [8]–[16]. In particular, wolf populations along the North Pacific Coast have previously been identified as morphologically [17], [18] and genetically distinct [10], [11], suggesting independent phylogeographic histories of coastal and continental lineages. Weckworth et al. [10], [11] demonstrated the distinctiveness of Alexander Archipelago and adjoining coastal (Alaska) wolves from adjacent continental populations and attributed diversification to barriers to gene flow through the coastal mountains. To the south of these Alaska populations, Muñoz-Fuentes et al. [15] attribute the genetic distinctiveness of coastal and island wolves from inland British Columbia (BC) wolves to the unique ecological and environmental characteristics of the region. To date, no genetic analysis has included both coastal Southeast Alaska and coastal BC wolves to assess their similarity. Nevertheless, both coastal BC and coastal Southeast Alaska wolves demonstrate life history characteristics distinct from inland continental populations [19]–[22].

Phylogeographic studies of multiple carnivore species found along the North Pacific Coast of North America reveal shared histories between Southeast Alaska and coastal British Columbia [5], [23], [24]. Given the new investigations that independently describe unique coastal wolves in either British Columbia or Southeast Alaska, a comprehensive analysis of the phylogeographic and population dynamics of both groups of coastal wolves is needed. Using mtDNA data, we augment previous analyses with a combined dataset of coastal BC and Southeast Alaskan specimens and include an expanded series of continental populations from interior Alaska, British Columbia and Yukon Territory to examine phylogenetic relationships, phylogeographic history, and the population dynamics of coastal wolves across their entire Northwest North American distribution.

## Methods

### Ethics Statement

This research was undertaken with approval from the National Park Service's Institutional Animal Care and Use Committee (protocol approval number NPS IACUC 2010-1).

### Sampling

**Figure 1:**
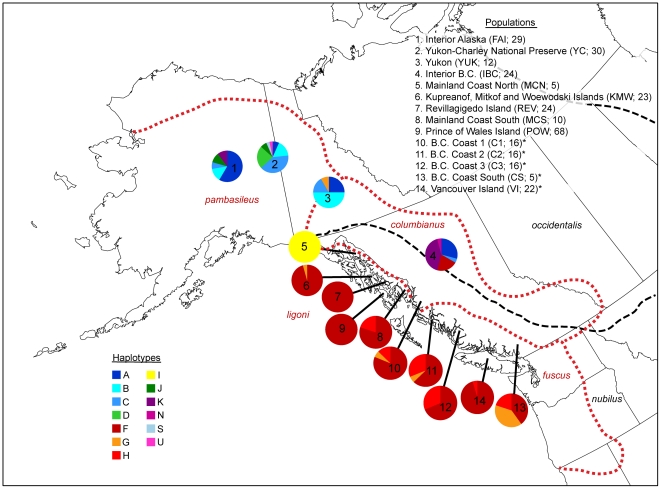
Map of sampling locations in Northwest North America. In the upper right, each population name is listed with its associated abbreviation and sample size. Asterisks indicate populations from Muñoz-Fuentes et al. [15]. Pie diagrams for each population indicate haplotype frequency, with color legend in the lower left. Black dashed lines indicate subspecies in the Pacific Northwest according to Nowak [33] and red dotted lines indicate the range of subspecies according to Hall and Kelson [32]. The 7 Southern United States (SUS) samples are not shown.

The sampling regime emphasized localities within continental and coastal Southeast Alaska and British Columbia (Figure 1), as described in Weckworth et al. [10], [11], including individual islands (REV) or island groups (KMW, POW) in the Alexander Archipelago; coastline of Southeast Alaska (MCN, MCS); interior Alaska (FAI); interior British Columbia (IBC); Yukon Territory (YUK). Eight populations, representing 193 individuals from Weckworth et al. ([11]; Text S1) were used in this study. We added 30 individuals from Yukon-Charley Rivers National Preserve (YC; Text S1), extending the previous continental sampling to the north. Additionally, we incorporated sequence data of 75 individuals from coastal British Columbia populations first presented in Muñoz-Fuentes et al. [15]; Vancouver Island (VI), three coastal populations in central BC (C1, C2, and C3), and one population in southern, coastal BC (CS) (Text S1). These sequences, along with sequences from pre-extirpated populations in the conterminous United States ([25]; Text S1) were obtained from GenBank. In total, the combined datasets yielded a sum of 310 individuals (including 30 previously unpublished samples from YC) representing 14 contemporary populations and 7 haplotypes from extirpated populations in the conterminous U.S. [25]. To accommodate differences in sequence length among these datasets, we reduced our original 613 bp segment of the mtDNA [11] to the overlapping 426 bp to match the coastal BC haplotypes from Muñoz-Fuentes et al. [15]. This subset of sequence includes the mtDNA tRNAs (91 bp) and portion of the control region (335 bp). Extraction and amplification protocols for the 30 novel individuals followed Weckworth et al. [11].

### Data Analysis

Phylogenetic relationships among haplotypes were examined by constructing a network using the median-joining method available in Network v.4.6 [26]. Arlequin v3.01 [27] was used to calculate haplotype and nucleotide diversity for each population. We generated pairwise Φ_ST_ statistics [28] in Arlequin to assess population differentiation. PASW Statistics 18.0 (SPSS Inc., Chicago, USA) was used to visualize the genetic relationships among populations with multidimensional scaling using pairwise Φ_ST_ estimates.

To test significant geographic divisions of hypothesized *a priori* subspecies groups, we used analyses of molecular variance (AMOVA [29],) in Arlequin [30]. This hierarchical analysis of variance partitions the total variance into covariance components due to differences among groups, among populations within groups and within populations. These calculations were performed using pairwise distances. In addition to the *a priori* Pacific Northwest and Alaskan subspecies hypotheses (i.e. [11], [31], [32]) and the broad-scale divisions suggested by the analysis of microsatellite loci (i.e., continental-coastal designations [10];), we also experimented with various *a posteriori* groups in AMOVA analyses. The *a posteriori* groups were suggested by the ambiguous placement of samples from British Columbia, analyses of nuclear DNA population trees, and Bayesian analyses of population structuring, as well as those suggested by geographical isolation (see below and [10]). We assumed that the best geographic subdivisions were significantly different from random distributions and had maximum among group variance (Φ_CT_ values). Thus, if there is concordance between the distribution of genetic subdivisions, and presumed subspecies delineations, values of Φ_CT_ should be significant, and larger than alternative groupings.

To investigate the distribution of genetic diversity over the contemporary samples, an *F*-statistics based spatial analysis of molecular variance (SAMOVA) was performed using SAMOVA 1.0 [33]. This method defines groups of populations that are geographically homogenous and maximally differentiated. The groupings that maximized *F*_CT_ while minimizing *F*_SC_ were assumed to be the most probable geographical subdivisions.

## Results

The genetic diversity described here is similar to the results of previously published studies (e.g. [11], [15]). Based on the shorter sequence length, we recorded 20 haplotypes across all individuals in the study (Figure 1) including haplotypes from extirpated regions (Not shown in Figure 1). There was one indel present, which was treated as a distinct character. The reduced sequence segment did not include a site that differentiated between two pairs of haplotypes (*A* and *M*, and *C* and *L*) from Weckworth et al. [11]; haplotype designations of *A* and *C* were included in this manuscript. Two previously undocumented haplotypes, *S* and *U*, were discovered in the Yukon-Charley population. This population also shares additional haplotypes with interior Alaska wolf populations (represented in this study by Fairbanks (FAI) area samples). Three haplotypes (*G*, *H*, *I*) are restricted to coastal British Columbia and Southeast Alaska. Haplotypes *G* and *H* are shared between coastal wolves in Southeast Alaska and coastal British Columbia. Haplotype *I*, however, is found only in Southeast Alaska wolves. The most common haplotype (*F*) found in the coastal region is also shared with wolves in interior British Columbia.

### Phylogeography

**Figure 2:**
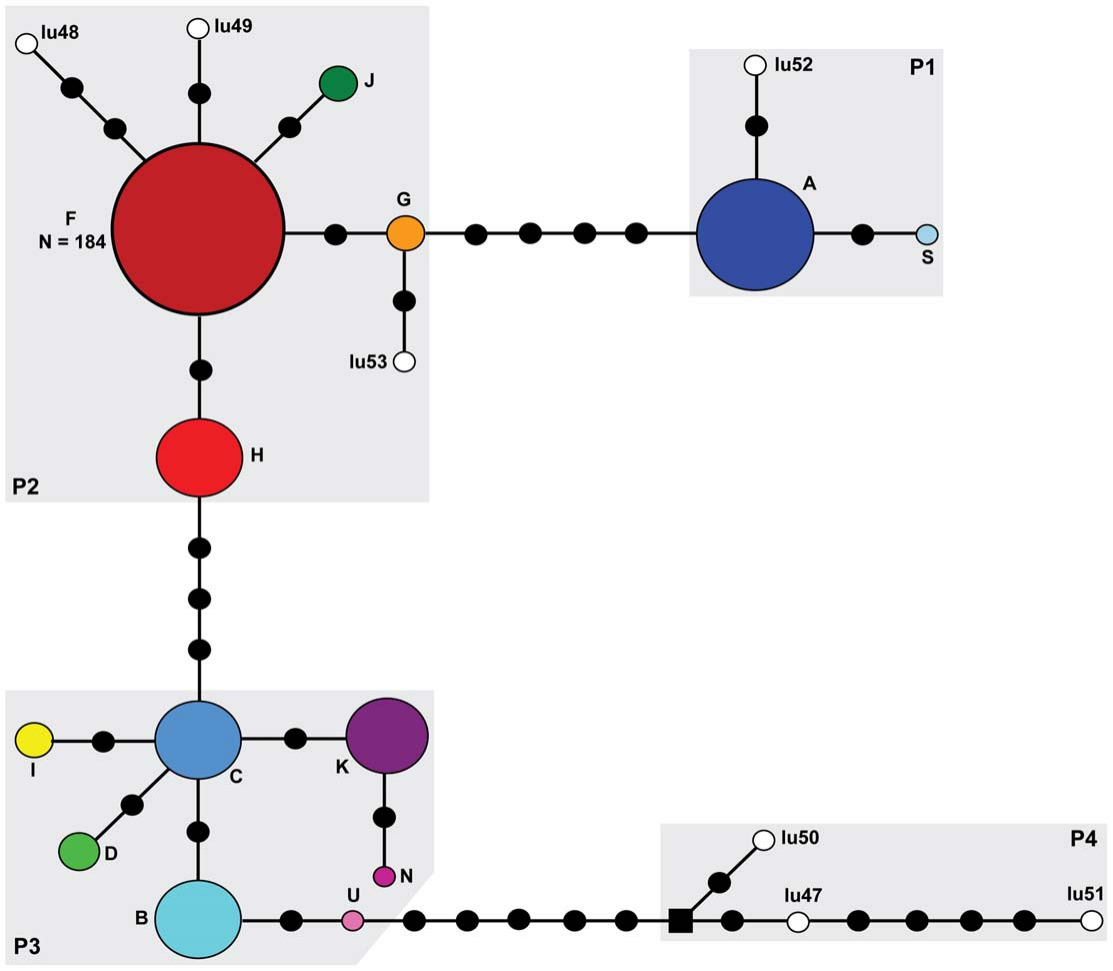
Haplotype network. Haplotype network for 20 haplotypes from 426 bp of mtDNA. Each black circle represents a single mutational change. The white circles indicate haplotypes from extirpated populations in the southern U.S [25]. The coloured circles and haplotype labels are consistent with Figure 1. The size of each circle is proportional to the observed frequency of a given haplotype. The maximum circle size is for N = 50, if N>50 the actual frequency is indicated. The black square represents a missing or hypothetical haplotype. Shaded regions define phylogroups 1, 2, 3 and 4 are labeled P1, P2, P3 and P4, respectively.

The haplotype network (Figure 2) includes 4 phylogroups in North America that are similar to those described in Weckworth et al. [11]. Phylogroup 1 includes the widespread continental haplotype (*A*) that was ubiquitous across northern Alaska and western Canada populations. Contemporary haplotypes in Phylogroup 1 (haplotypes *A* and *S*) have a distribution corresponding to *C. l. occidentalis*. However, the addition of the *lu52* haplotype from a historical specimen collected in Oklahoma [25] represents *C. l. nubilus*, and is not consistent with Nowak's [32] proposed taxonomy.

Coastal populations were predominately found in Phylogroup 2, which included haplotype *F*, present in 184 individuals from Southeast Alaska, and coastal and interior BC. This phylogroup also included haplotype *H* which is restricted to coastal BC and the southern mainland coastal area in Southeast Alaska. Haplotype *G* is shared between the North Pacific Coast and the Yukon, corroborating the southern refugial origin of some individuals [11], or reflecting the lack of sorting of ancestral genetic variation. With the exception of the YUK individual, and possibly the BC individuals, samples were within the geographic range of *C. l. nubilus* ([32]; samples from BC were collected at or near the boundary between *C. l. nubilus* and *C. l. occidentalis*). The presence of haplotypes from extirpated southern populations (*lu48*, *lu49*, *lu53*) in Phylogroup 2 (Figure 2) indicates a wider geographic distribution of Phylogroup 2 through the conterminous U.S., and supports the assertion of a southern refugial source for coastal wolves [10], [11]. Due to the loss of informative sites with the truncation of sequences from 611 to 426 bp, coastal wolves are not clustered in a single phylogroup (haplotype *I* is in Phylogroup 3) as found by Weckworth et al. [11].

Phylogroup 3 included haplotypes found within interior Alaska and the Yukon (*D*, *K*, and *C*). However, the displaced haplotype *I*, which is only found in one population in Southeast Alaska, is found within this phylogroup, which would suggest mixed refugial origins of coastal wolves, or contemporary gene flow between interior and coastal wolves. Individuals with haplotypes in phylogroup 3 (except for haplotype *I*) are within the defined range of *C. l. occidentalis*. Phylogroup 4 represents a distinct southern refugial group identified by Leonard et al. [25] as well as the endangered Mexican wolves (*C. l. baileyi*), and is diverged from other North American wolves (Figure 2).

### Population Structure

Population information for the haplotypes from extirpated populations is not available and consequently these sequences were not included in AMOVA analyses. When populations were divided into coastal and continental groups [10], AMOVA results (Table 1, Model A) indicate that 50.7% of all genetic variation distinguished geographic groups (*P* = 0.002), 34.1% of variation was apportioned within populations (*P*<0.0001), and 13.0% was relegated among populations within groups (*P*<0.0001). Model A partitions the populations into two groups that correspond to either the distribution of *C. l. nubilus* or *C. l. occidentalis* [32], testing the validity of the two subspecies. Transferring coastal BC populations from the coastal group into their own distinct group (i.e. consistent with three subspecies, adding BC coastal as *C. l. fuscus*) results in a reduction of Φ_CT_, although the value is still significant (Table 1, Model B). Model C incorporates coastal BC populations with interior Alaska and Canada populations to test whether or not the coastal BC populations should be included in *C. l. occidentalis* [32]. Model D is a general AMOVA test within *C. l. occidentalis* splitting the populations into three groups: coastal wolves, interior Alaska wolves, and interior Canada wolves. This test result was also significant (Table 1). Finally, Model E tests subspecies designations applied by Hall and Kelson [31] to wolves of the North Pacific and Alaska (e.g., *C. l. ligoni* applied to wolves of the Alexander Archipelago and southeast mainland, *C. l. fuscus* for coastal BC, *C. l. pambasileus* applied to wolves elsewhere in Alaska, and C. *l. columbianus* describing wolves of the Yukon and interior BC). Overall, the results of the AMOVA analysis suggest that *C. l. ligoni* is genetically distinct from *C. l. occidentalis* when coastal British Columbia is included.

**Table 1:** AMOVA results.

Model	Hypothesized groupings	Φ_SC_	Φ_ST_	Φ_CT_	% among groups	p Φ_CT_
A	[KMW, MCN, MCS, POW, REV, VI, CBC, CS] [IBC, YC, FAI, YUK]	0.300	0.655	0.507	50.69	0.00196
B	[KMW, MCN, MCS, POW, REV] [VI, CBC, CS] [IBC, YC, FAI, YUK]	0.331	0.613	0.433	42.21	0.00782
C	[KMW, MCN, MCS, POW, REV] [VI, CBC, CS, IBC, YC, FAI, YUK]	0.448	0.605	0.287	28.56	0.05376
D	[KMW, MCN, MCS, POW, REV, VI, CBC, CS,] [YC, FAI] [IBC, YUK]	0.322	0.628	0.451	45.09	0.00391
E	[KMW, MCN, MCS, POW, REV] [VI, CBC, CS] [YC, FAI] [IBC, YUK]	0.343	0.582	0.365	36.47	0.01760

Analysis of molecular variance for five *a posteriori* models of groupings according to different subspecies designations previously identified using morphological data.

Population subdivision based on mtDNA was calculated using SAMOVA. The results indicated that genetic differentiation among groups was maximized at six groups (*F*_CT_ = 67.06) while differentiation between populations within groups dropped below zero (*F*_SC_ = −0.15). All of the continental populations and MCN were each in a group by themselves. The sixth group was composed of all the coastal populations (except for MCN).

**Figure 3:**
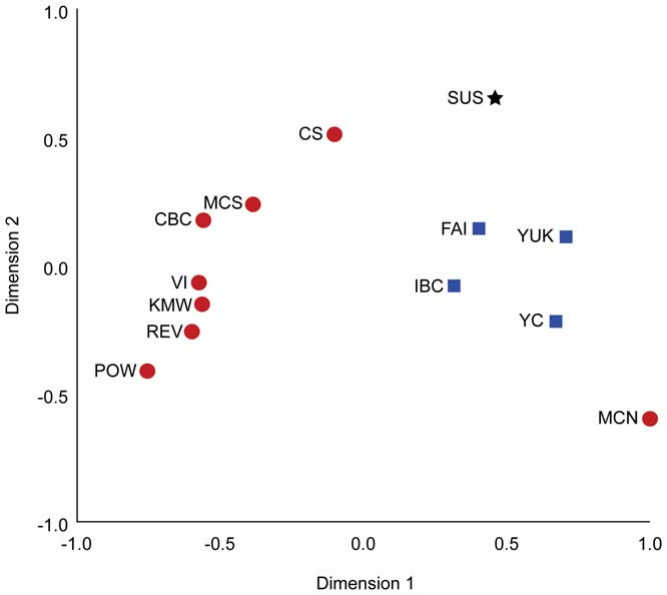
Multi-dimensional scaling plot. Non-metric multi-dimensional scaling plots of populations based on pairwise Φ_ST_ results. Red circles are coastal populations, blue squares are continental, and the black star represents the extirpated conterminous U.S haplotypes. Population abbreviations are from Figure 1.

Population pairwise Φ_ST_ values indicate geographic structuring across the landscape for many populations. Pairwise estimates of Φ_ST_ (Table 2) are consistent with F_ST_ in microsatellites [10] and significantly correlated (Mantel Test, p = 0.0018), with genetic distances highest between coastal and continental population comparisons [11]. Coastal BC populations in proximity to each other (C1, C2, and C3) were not significantly different from each other (using the 426 bp sequence in this study) and therefore, were placed together as a single group (CBC) for pairwise Φ_ST_ results (Table 2). All Southeast Alaska populations other than MCN (which has a unique haplotype) were not significantly differentiated from Vancouver Island, and the MCS population (southern Southeast Alaska) was not significantly differentiated from any of the coastal BC populations. Other Southeast Alaska island populations are significantly differentiated from one another as well as from all coastal British Columbia populations (Table 2). Multi-dimensional scaling ordinations of the spatial patterns of genetic variation among the populations (Figure 3) show a distinct separation of coastal and continental populations, with the exception of MCN.

**Table 2:** Φ_ST_ Population pairwise comparisons.

	VI	CBC	CS	IBC	YC	FAI	YUK	KMW	MCN	MCS	POW	REV
**CBC**	***0.084***											
**CS**	***0.396***	0.129										
**IBC**	***0.442***	***0.494***	***0.248***									
**YC**	***0.673***	***0.691***	***0.523***	***0.163***								
**FAI**	***0.534***	***0.590***	***0.336***	***0.121***	***0.351***							
**YUK**	***0.719***	***0.732***	***0.432***	0.086	***0.116***	***0.129***						
**KMW**	0.000	***0.130***	***0.380***	***0.451***	***0.681***	***0.536***	***0.724***					
**MCN**	***0.985***	***0.913***	***0.904***	***0.346***	***0.313***	***0.520***	***0.451***	***0.985***				
**MCS**	0.063	0.054	0.122	***0.330***	***0.581***	***0.444***	***0.584***	0.154	***0.949***			
**POW**	0.061	***0.241***	***0.775***	***0.637***	***0.808***	***0.701***	***0.872***	0.056	***1.000***	***0.472***		
**REV**	0.004	***0.149***	***0.551***	***0.465***	***0.692***	***0.552***	***0.746***	0.002	***1.000***	0.248	0.000	
**SUS**	***0.634***	***0.708***	***0.361***	***0.370***	***0.501***	***0.391***	***0.359***	***0.637***	***0.544***	***0.503***	***0.817***	***0.656***

Pairwise population comparisons calculating Φ_ST_. Bold-italicized numbers indicate significant p-values (α = 0.05). Abbreviations as per text and Figure 1 (CBC = coastal British Columbia and is the combined data of C1, C2 and C3).

## Discussion

Independent studies previously demonstrated that coastal lineages of wolves in Southeast Alaska and coastal British Columbia [10], [11], [15] are each distinct from other North American continental wolves. Our analyses are the first to include specimens from both coastal Alaska and coastal British Columbia and indicate a close evolutionary relationship between all coastal wolves relative to continental wolves. Wolves in western North America mirror a phylogeographic pattern of distinctive coastal and continental lineages repeatedly identified in other mammals such as black bears [4], marten [24], [34], [35], flying squirrels [36], [37], deer mice [3] as well as multiple plant species [38]. Coastal lineages tend to share a similar phylogeographic history, as reflected in mitochondrial genomes, which are divergent from nominally conspecific continental populations. In those cases where independent nuclear markers have been assessed (e.g. [10], [35]), this coastal/continental divergence has been corroborated.

Although both coastal British Columbia and Southeast Alaska wolves share similar evolutionary histories, genetic diversity varies among the regions. Haplotype diversity was similar across the combined regions, with the exception of a fourth haplotype (*I*) that was unique to five individuals from the Juneau region of the northern mainland coastal area (MCN) of Southeast Alaska. Populations of island wolves in coastal BC generally possess multiple haplotypes, whereas most island wolves in Southeast Alaska were monotypic for the common coastal haplotype (*F*), suggesting that either gene flow between mainland coastal and island wolves is higher in BC than Southeast Alaska, or that island wolves in Southeast Alaska have been subjected to extreme genetic drift, perhaps due to small founding populations or subsequent bottlenecks. Weckworth et al. [10] analyzed the same individuals using hypervariable nuclear microsatellites and found no evidence of recent or historic bottlenecks across any of the Southeast Alaska populations. However, the methods they used to detect bottlenecks have decreased statistical power if a severe bottleneck (e.g. the population was decreased to fewer than 25 effective breeders [N_e_]) occurred more than 100 generations (ca. 4N_e_) ago [39].

Two of the four haplotypes identified in the coastal lineage of wolves (*F* and *G*) were found in continental populations. Haplotype *F* was found in 87% of all coastal individuals, and was identified in some continental populations (e.g. interior BC; this study; haplotype equivalent lu38 in [15]) suggesting gene flow from coastal populations into adjacent interior BC populations. Haplotype *G* was found in only 6 individuals here, but the equivalent haplotype has been identified broadly and predominantly across continental populations (*lu32*, [15]) and may indicate gene flow into coastal populations. Conversely, these patterns may simply correspond to incomplete lineage sorting since expansion from refugial populations, or historic gene flow, as assessments of nuclear microsatellite data indicate little contemporary gene flow between coastal and continental populations [10]. Further analyses using microsatellite or other nuclear loci should be extended to include the entire coastal wolf distribution and adjacent continental populations and, combined with next generation sequencing, would further clarify contemporary levels of genetic exchange and the evolutionary history of these populations.

Muñoz-Fuentes et al. [15] and Weckworth et al. [10] cite the distinctive ecological characteristics of the coastal biome of the North Pacific Coast region and geographic isolation, respectively, as the major mechanisms for diversification of coastal wolves. Weckworth et al. [11] also provide evidence that wolves in northern latitudes in North America emerged from different refugia. After colonization, ecology and isolation, as mechanisms of diversification, are not mutually exclusive. The ecological argument for parapatric divergence as the contemporary mechanism for reinforcing the separation of coastal and continental BC populations [15] is particularly compelling given the demonstrated ability of wolves to successfully colonize many different terrestrial ecosystems. Studies of other taxa in the Pacific Northwest, particularly Southeast Alaska, have concluded that coastal and continental lineages evolved in separate refugia during the Pleistocene (black bears, [4], [40]; marten, [5], [24]). Unlike wolves, some of these species are arguably more narrowly restricted to particular habitats, but, in several cases the continental lineage has expanded into sympatry with representatives of the coastal lineage [4], [5], [24], [35]. Intense territoriality of wolves, combined with their complex social hierarchies, may curtail the establishment of dispersing continental individuals, and may have consequently helped to reinforce parapatric divergence between coastal and continental wolf populations.

The continental populations studied here cover largely intact habitat across a nearly contiguous range and demonstrate patterns of genetic diversity that suggest few barriers to gene flow. In contrast, coastal populations of Southeast Alaska are distributed across a naturally fragmented region. Anthropogenic activities such as logging and road building have increased access and trapping, and the illegal hunting of these populations, particularly in Southeast Alaska [41], [42]. Increased mortality may result in the continued loss of genetic diversity in these coastal wolves, or the breakdown of reinforcement mechanisms that have largely prevented introgression with continental wolves [43].

Weckworth et al. [11] propose that wolves in Southeast Alaska originated from a southern refugium, and represent the last remnants of genetic diversity from extirpated historic populations of wolves once found in the conterminous United States. Multiple subspecies of wolves were described for the Pacific Northwest; *C. l. ligoni* in Southeast Alaska, *C. l. fuscus* of coastal BC, Washington and Oregon (now only extant in coastal BC), and *C. l. crassodon* on Vancouver Island [17], [18]. Nowak's [32] revision of subspecific designations in wolves subsumed these coastal subspecies into a single widespread subspecies, *C. l. nubilus*, which extended to wolf populations across most of the conterminous US and into eastern Canada. Subsequent molecular perspectives [10], [11], [15] revealed distinctive coastal wolves and our analyses support the distinctiveness of coastal wolves as a single phylogeographic lineage along the North Pacific Coast that would encompass *C. l. ligoni*, *C. l. fuscus* and *C. l. crasodon*.

### Conservation Implications

Coastal wolves have been described as distinct Management Units [15] in Canada, following Moritz [44], but currently have no special management consideration in Southeast Alaska [11]. Given the imprecision of population estimates for coastal Alaska, legal and illegal harvest [42], and the apparent genetic isolation of coastal wolf populations as a whole [10] [11], special caution is warranted in evaluating the consequences of “expected” population declines on wolves in Southeast Alaska, specifically (62 Federal Register 46710). More generally these preliminary molecular surveys call for a re-evaluation of geographic variation over the entire range of coastal wolves.

*Canis lupus* is listed as vulnerable across its global range (North America, Eurasia, and the Middle East) [45]. The coastal wolves analyzed here have previously been identified as a subspecies (*C. l. ligoni*) restricted to temperate rainforests of coastal Southeast Alaska and British Columbia. In the U.S., 80% of the terrestrial biome is managed by the U.S. Forest Service (Tongass National Forest). These coastal wolves are considered a species [46] or subspecies [47], [48] of concern in the U.S., and a Management Indicator Species for the Tongass National Forest [49]. In 1997 the U.S. Fish and Wildlife Service (FWS) was petitioned to list the coastal wolves of the Alexander Archipelago as a threatened species under the Endangered Species Act based on wolf viability in response to timber practices on the Tongass and associated prey depletion and increased access for wolf trappers and hunters (62 Federal Register 46710). FWS issued a “not warranted” finding. Although FWS expected the Alexander Archipelago wolf populations to decline, the agency did not consider the population to be in danger of extinction in the foreseeable future because they “expect the population decline to stop at an acceptable level.” Additionally, wolves are known to persist at low numbers in healthy populations and to be resilient to the activities of man because of their high reproductive rate and high dispersal capability (62 Federal Register 46710).

The high dispersal capabilities cited by the FWS presumably suggest that recruitment of wolves from outside of Southeast Alaska would mitigate declining populations and loss of genetic diversity. This study and previous work [10], [11], [15], consistently indicate minimal gene flow between coastal and continental populations. Instead, a more detailed understanding of recruitment and gene flow between coastal Canadian and coastal Alaskan populations appears to be essential for effective trans-boundary management. However, no international agreements currently support such analyses. Although wolves can persist successfully at low population densities, theoretical modeling [50], research on other restricted carnivore populations [51], [52] and empirical data from populations of other island species [53], [54] suggest that prolonged bottlenecking will result in loss of genetic variation, especially in areas at already low levels of genetic diversity.

Previous studies focusing on wolves in the continental United States found minimal variation among populations (e.g. [25]). In high latitude ecosystems, however, it is clear that coastal and continental wolf populations demonstrate both significant regional and inter-population differentiation [10], [11, this study]. It is therefore not surprising that mtDNA analyses of a previously unassayed continental wolf population (Yukon-Charley Rivers National Preserve) would uncover significant population differentiation and novel haplotypes. It is possible, and certainly testable, that these novel haplotypes occur in other unassayed populations, including those in the adjacent Yukon Territory of Canada.

Habitat of wolves of the Yukon-Charley National Preserve is protected by the National Park Service, which is explicitly mandated to assess and maintain variability of wildlife populations [55], [56]. Nevertheless, there are concerns about the long-term maintenance of genetic variation in this population as the 15–20% mortality due to subsistence, sport hunting and trapping on the national preserve [57] is currently being augmented by intensive predator control efforts intended to reduce wolf predation on caribou herds by reducing wolf population numbers by 60–80% [58]. Because packs whose territories include Yukon-Charley also travel outside of the preserve, including into Yukon Territory [57], [59], their social and genetic structure [60] may be impacted by management prescriptives fostered by state/provincial and national management agencies of two countries.

### Conclusions

The coastal wolves in Southeast Alaska and coastal British Columbia represent a distinct portion of the genetic diversity for all wolves in North America. Moreover, increased sampling across continental populations will reveal additional variation as exhibited in Yukon-Charley wolves. Given the intensity of current efforts to control wolves in many areas, our assessment of phylogeographic structure across the North Pacific region suggests that a much more refined understanding of genetic variation is needed to ensure the persistence of this high profile carnivore throughout the region.

Wolves are a trans-boundary species and, as demonstrated here, exhibit metapopulation dynamics that encompass habitats in both Canada and the US. This necessitates increased international cooperation for wolf management and conservation. The success of such geographic integration of management programs has been demonstrated for other taxonomic groups, particularly migratory birds [61]. The legal framework for international collaboration exists in the “Framework for Cooperation between the US Department of the Interior and Environment Canada in the Protection and Recovery of Wild Species at Risk” signed by the governments of the US and Canada in 1997 [62], and through the government-funded North American Commission for Environmental Cooperation. These agreements recognize wolves as an international species of conservation concern, but are focused primarily on the conterminous US where wolf populations were extirpated by the mid-20^th^ century. The extension of such international collaborations to include Alaska, British Columbia and Yukon Territory will encourage recognition of the importance of wolf populations in regions outside the extirpation zone, and support the establishment of trans-boundary management plans that maintain the important ecological and genetic diversity of these shared northern populations.

## Supporting Information

Text S1 **Sample accession numbers.** Description of *Canis lupus* GenBank mitochondrial DNA sequences and the location of original tissue sample if known. Listed by original publication are populations (abbreviations as given in text), GenBank accession numbers and when available, in corresponding order, the voucher number from either University of Alaska Museum of the North (UAM) or Museum of Southwestern Biology (MSB).
(DOCX)
