# Supplementary material for: Going Coastal: Shared Evolutionary History between Coastal British Columbia and Southeast Alaska Wolves (Canis lupus)
Source: PLoS One. 2011 May 4;6(5):e19582. doi: 10.1371/journal.pone.0019582 (PMC3087762; doi:10.1371/journal.pone.0019582)
Supplement: Text S1 — Sample accession numbers. Description of Canis lupus GenBank mitochondrial DNA sequences and the location of original tissue sample if known. Listed by original publication are populations (abbreviations as given in text), GenBank accession numbers and when available, in corresponding order, the voucher number from either University of Alaska Museum of the North (UAM) or Museum of Southwestern Biology (MSB). (DOCX) [file pone.0019582.s001.docx]

*Weckworth et al. 2010*: **FAI**, GQ376249-GQ376277, UAM (28836, 28847, 28852, 28806, 28857, 28862, 28867, 28910, 28816, 34400, 45007, 49416, 49424, 49429, 63739, 63750, 70583, 28842, 28811, 28901, 28878, 28795, 44996, 47441, 47454, 52181, 44990, 45002, 49434); **IBC**, GQ376203-GQ376226, UAM (36516, 36535, 36537, 36540, 36541, 36543, 36525, 36528, 36542, 36545, 36532, 36519, 36520, 36533, 36534, 36539, 36544, 36522, 36524, 36527, 36529, 36536, 36518, 36521); **KMW,** GQ376376-GQ376398, UAM (24887, 24889, 24890, 24891, 24977, 47419, 47421, 47423, 47424, 47425, 51723, 44541, 51655, 69232, 69233, 69234, 49838, 49840, 24910, 24914, 47422, 21440, 47420); **MCN**, GQ376399-GQ376403, UAM (36294, 66897, 24888, 24895, 24897); **MCS,** GQ376404-GQ376413, MSB (152778, 151939, 151886, 151894, 151920, 151924) UAM (24893, 24708, 24711, 24707); **POW**, GQ376414-GQ376481, MSB (151888, 151885, 151896, 151919) UAM (24911, 24903, 30127, 30128, 24710, 49368, 49371, 49373, 49375, 49376, 49377, 49380, 30324, 30325, 30326, 30328, 30330, 73552, 30132, 24917, 24918, 49374) MSB (151933, 151935, 151940, 151890, 151898, 151941) UAM (30329, 30126, 24103, 24574, 24108, 24111, 21442, 69509, 21441, 76285, 24107, 76376, 76377, 24765, 24919) MSB (151887, 151895, 151897, 151922, 151882) UAM (51659, 47184, 47185, 44552, 51660, 51661, 47186, 44551, 51662, 49499, 47187, 47188, 51658, 49500, 44550, 44553); **REV,** GQ376482-376505, UAM (73553, 30425, 30680, 30679, 29155, 30131) MSB (152771, 152772, 152775, 152776, 152777, 152779, 152780, 152783, 151932, 151934, 151936-151938, 151889, 151891, 151923, 151927, 151883); **YUK,** GQ376364-GQ376376, UAM (73513, 73523, 73525, 73512, 73514, 73515, 73516, 73518, 73519, 73522, 73517, 73520).

*Muñoz-Fuentes et al. 2009*: **VI**, **C1**, **C2**, **C3**, **CS,** FM201598-FM201777 (published information does not identify individual GenBank accession numbers to population. In our analyses we used cited haplotype names and their proportional numbers in each population.)

*Leonard et al. 2005*: **SUS,** AY812732-AY812738

*This study*: **YC,** JF311405-311434, UAM (63137, 63109, 63146, 63135, 62791, 62793, 62788, 104774, 63149, 63144, 63117, 63145, 62717, 62716, 63141, 62715, 63136, 62794, 63114, 63781, 62783, 62779, 62792, 63129, 62778, 63148, 62718, 62735, 104773, 63147).
